# Cluster of Ebola Cases Among Liberian and U.S. Health Care Workers in an Ebola Treatment Unit and Adjacent Hospital — Liberia, 2014

**Published:** 2014-10-17

**Authors:** Joseph D. Forrester, Jennifer C. Hunter, Satish K. Pillai, M. Allison Arwady, Patrick Ayscue, Almea Matanock, Ben Monroe, Ilana J. Schafer, Tolbert G. Nyenswah, Kevin M. De Cock

**Affiliations:** 1 Epidemic Intelligence Service, National Center for Emerging and Zoonotic Infectious Disease; 2 Division of Preparedness and Emerging Infections, National Center for Emerging and Zoonotic Infectious Disease; 3 Division of High-Consequence Pathogens and Pathology, National Center for Emerging and Zoonotic Infectious Disease; 4 Division of Epidemiology, Analysis, and Library Services, Center for Surveillance, Epidemiology, and Library Services, CDC; 5 Liberian Ministry of Health and Social Welfare; 6 CDC Kenya; 7 Division of Global HIV/AIDS, Center for Global Health, CDC

The ongoing Ebola virus disease (Ebola) epidemic in West Africa, like previous Ebola outbreaks, has been characterized by amplification in health care settings and increased risk for health care workers (HCWs), who often do not have access to appropriate personal protective equipment. In many locations, Ebola treatment units (ETUs) have been established to optimize care of patients with Ebola while maintaining infection control procedures to prevent transmission of Ebola virus. These ETUs are considered essential to containment of the epidemic. In July 2014, CDC assisted the Ministry of Health and Social Welfare of Liberia in investigating a cluster of five Ebola cases among HCWs who became ill while working in an ETU, an adjacent general hospital, or both. No common source of exposure or chain of transmission was identified. However, multiple opportunities existed for transmission of Ebola virus to HCWs, including exposure to patients with undetected Ebola in the hospital, inadequate use of personal protective equipment during cleaning and disinfection of environmental surfaces in the hospital, and potential transmission from an ill HCW to another HCW. No evidence was found of a previously unrecognized mode of transmission. Prevention recommendations included reinforcement of existing infection control guidance for both ETUs and general medical care settings,* including measures to prevent cross-transmission in co-located facilities.

## Investigation

On July 26, 2014, Liberian Ministry of Health and Social Welfare was informed of a laboratory-confirmed case of Ebola in an HCW at an ETU located adjacent to a general hospital (hospital A) in Monrovia, Liberia; in the following 24 hours CDC was informed of two additional HCW cases at the same ETU. Concern among HCWs and patients about the possible risk for Ebola transmission resulted in suspension of hospital and ETU operations. During July 27–31, CDC conducted a rapid evaluation to identify additional cases among HCWs and possible sources of exposure at the request of the Liberian Ministry of Health and Social Welfare and the humanitarian relief organizations involved in ETU and hospital A operations. Given time constraints in an evolving, somewhat chaotic epidemic environment, evaluation methods included unstructured in-person and telephone interviews with the infected HCWs, staff members and volunteers at the ETU and hospital A, and administrators, as well as onsite visits to hospital A and the ETU (at both its initial and relocated sites) (Figure). Employee work schedules were reviewed when available. Exposure risk to HCWs outside of the work environment at the ETU or hospital A were assessed through interview when possible.

Cases of Ebola were categorized as suspected, probable, or confirmed; this was consistent with the CDC Ebola virus disease case definitions in use in the field during the investigation. A suspected case was defined as fever and three or more additional symptoms (intense fatigue, myalgia, headache, nausea, difficulty in breathing or swallowing, hiccups, abdominal pain, vomiting, and diarrhea); fever with signs and symptoms of hemorrhage, or any unexplained death. A probable case was an illness meeting the suspected case definition in a person who had contact with a person with a confirmed or probable case in the past 3 weeks, or had at least fever and contact with a person with a confirmed or probable case in the past 3 weeks. A confirmed case was a suspected or probable case with laboratory evidence of Ebola virus infection by reverse transcription–polymerase chain reaction at the National Reference Laboratory in Liberia.

## Findings

Hospital A is a private community hospital with approximately 150 to 200 inpatient admissions per month; its predominant function is provision of general medical care. Because of its proximity to the ETU (at the time, the only ETU in Monrovia), hospital A functionally served as a triage point for patients with suspected Ebola. Protocols for diverting Ebola patients to the ETU from hospital A’s emergency department included a triage area at the entrance to the emergency department; patient screening for risk factors for Ebola; and direct transfer of suspected, probable, and confirmed cases.

Five HCWs (three Liberian nationals and two U.S. nationals) who worked at the ETU, hospital A, or both, were identified as being infected with Ebola virus during July 14–July 29 (HCWs A, B, C, D, and E); two died from their Ebola virus infection. Work responsibilities and clinical features of the five HCWs varied (Table). No unprotected exposures to Ebola patients or contaminated surfaces were reported by HCWs in the ETU (staff reported adherence to personal protective equipment guidelines consistent with job duties in the ETU) (1). Information about exposure outside of work to persons with Ebola could not be determined for the three HCWs (A, D, and E) who died or were otherwise unavailable at the time of evaluation.

Three findings from the evaluation of the health care environment and health care practices were identified as opportunities for transmission of Ebola virus: First, at the hospital A emergency department, failure to identify patients with Ebola promptly resulted in delayed transfer to the ETU (by several hours to >1 day); in one case, a patient with undiagnosed Ebola died in the emergency department, potentially exposing HCWs. Second, daily fever and symptom monitoring was not routinely performed on the staff at the ETU or hospital A; a HCW working in these areas could become infected, yet go undetected. Third, all ETU and hospital A staff had access to hospital A facilities, including eating areas, showers, bathrooms, and work stations and direct, physical contact between staff members in these common areas was reported; transmission between an infected, but undetected, coworker could occur.

Regarding the transfer of Ebola patients from the hospital A emergency department to the ETU, the investigation revealed that on June 26 one confirmed patient and on July 14 one confirmed and one probable patient (none part of the five-HCW cluster) were treated for other diseases in the hospital A emergency department while their Ebola remained unrecognized, leaving bodily fluids on surfaces in the emergency department that required cleaning and disinfection.

### Discussion

Despite the temporal and geographic clustering of the five HCWs with Ebola, no common source exposure or chain of transmission to explain all five cases was identified. Because persons being treated for other diseases in the emergency department of hospital A (adjacent to the ETU) had undiagnosed Ebola, patients or coworkers in this hospital or the immediate surrounding area might have been at higher risk. Specifically, three opportunities for exposure consistent with known Ebola virus transmission modes were identified in this HCW cluster: 1) HCW exposures to undetected Ebola patients treated before their diagnosis in hospital A, 2) inadequate use of personal protective equipment during cleaning and disinfection of grossly contaminated surfaces in hospital A, and 3) exposure of noninfected HCWs to infected HCWs in the ETU or hospital A. Three infected HCWs (B, C, and D) participated in activities that included spraying disinfectant in the ETU or hospital A; however, the risk for exposure to Ebola virus from these activities could not be assessed during this investigation. There were no self-reported, unprotected exposures to Ebola patients or contaminated materials in the ETU. Staff reported adherence to personal protective equipment use consistent with job duties in the ETU (1). Based on interviews, protection against exposure to Ebola virus might have been less stringent outside of the ETU than inside it. Clinical and cleaning and disinfection activities in the adjacent hospital and triage area of hospital A potentially served as unrecognized, but nonetheless high risk, exposures. Shared facilities and physical contact with coworkers could have resulted in transmission of Ebola virus if a coworker was infected, but not diagnosed. None of the information collected suggested a mode of Ebola virus transmission that had not previously been described.

The findings in this report are subject to at least three limitations. First, interviews were not performed in a standardized format, so formats of responses varied. Second, two HCWs in this cluster had died before the start of the investigation, and one was unable to be interviewed, so exposure history in these three persons was obtained through interviews with coworkers or administrators. Finally, exposure history for these three persons was based on postevent interviews in a chaotic and stressful environment; therefore, recall might be incomplete.

Several action items were identified for public health intervention. All hospitals in epidemic areas should be considered as sites where Ebola patients might come for medical care and should ensure patients can be promptly identified and safely isolated (2). HCWs working in epidemic areas should maintain a high index of suspicion regarding patients who have any of the signs or symptoms of Ebola.† All HCWs should be trained to recognize signs and symptoms of Ebola, have personal protective equipment§ available that is suitable for protecting themselves from transmission of Ebola virus, and be trained in its use. Separation of ETUs from hospitals, including designating trained HCW staff to provide health care only at the ETU, and provision of independent facilities such as restrooms, eating, and work areas, could minimize the opportunities of HCW exposure to Ebola virus, as suggested by recent recommendations (1,2). Daily monitoring for signs and symptoms of Ebola, such as fever screening, could improve early detection and isolation of an Ebola virus–infected HCW. A strict “no touching” policy (1) among HCWs as advocated by Médecins Sans Frontières could reduce the opportunity for an infected, yet undiagnosed HCW to transmit Ebola virus to a coworker. Finally, four of five HCWs in this cluster worked commonly or exclusively at night; fatigue and reduced levels of supervision might contribute to suboptimal adherence to recommended preventive measures.

Rapidly identifying and isolating patients with Ebola is essential to preventing further transmission. ETUs are usually established in close collaboration with international health care organizations. Ebola virus infection of HCW staff members working at, or associated with, an ETU can undermine community confidence in the health care system, create new opportunities for ongoing transmission, and reduce an already insufficient clinical workforce. Preventing exposures of HCWs and reducing the risk for Ebola virus infection of HCW must continue to be a high priority to halt transmission of Ebola and maintain adequate care for Ebola patients.

What is already known on this topic?

The Ebola virus disease (Ebola) epidemic in West Africa has been characterized by amplification in health care settings and increased risk for health care workers (HCWs). Ebola treatment units (ETUs) have been established to optimize care of patients with Ebola while maintaining infection control procedures to prevent transmission of Ebola virus and protect HCWs. These ETUs are considered essential to containment of the epidemic.

What is added by this report?

Five cases of Ebola among HCWs at an ETU and an adjacent hospital in Monrovia, Liberia, did not have an identifiable common source of exposure or chain of transmission. However, opportunities existed for transmission of Ebola virus to HCWs in this cluster, including HCW exposure to unrecognized, infected patients outside of the ETU, inadequate use of personal protective equipment during cleaning and disinfection of environmental surfaces in hospital A, and potential transmission from an ill HCW to another HCW in the ETU or hospital A. No evidence was found of any previously unrecognized mode of transmission.

What are the implications for public health practice?

Health care workers in ETUs who have clinical, cleaning, or disinfection responsibilities in other settings might be exposed to infected persons or contaminated surfaces in those settings. Hospital emergency departments should be alert to quickly recognize and isolate persons with suspected Ebola. Appropriate infection control precautions and personal protective equipment should be available.

## Figures and Tables

**FIGURE:**
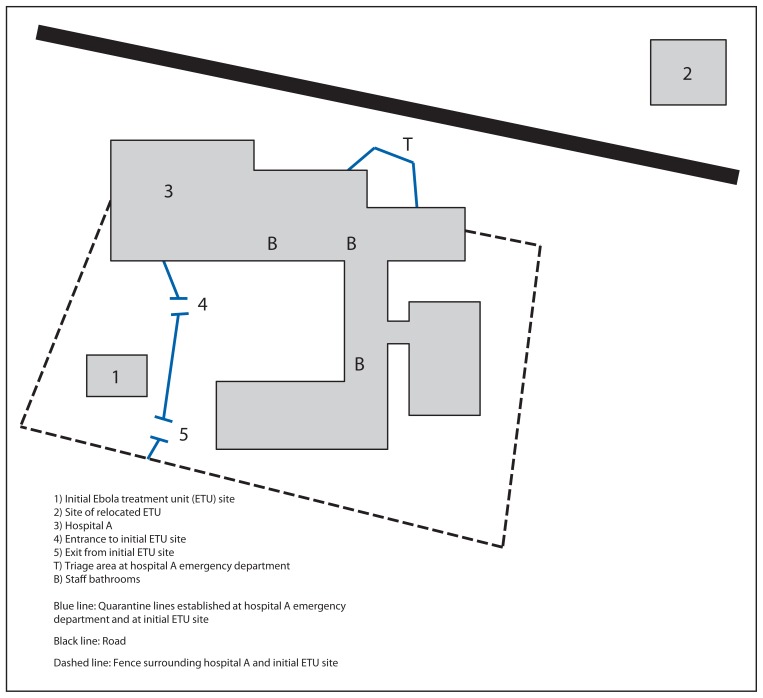
Location of hospital A and adjacent Ebola treatment units* — Monrovia, Liberia * The ETU was initially located on the grounds of hospital A (1) after opening during the second wave of the Ebola epidemic in late spring 2014. On July 20, 2014, the ETU was moved to a facility (2) approximately 100 meters (328 feet) away.

**TABLE:** Work responsibilities and clinical information for five health care workers (HCWs) who became infected with Ebola virus while working in an Ebola treatment unit (ETU) or an adjacent general hospital (hospital A) — Monrovia, Liberia, July 2014

Work responsibilities/Clinical information	HCW A	HCW B	HCW C	HCW D	HCW E
Work location	Hospital A ED	ETU and hospital A ED triage area	ETU and hospital A ED triage area	ETU (hospital A ED triage area: unknown)	Hospital A ED
Work shift; shift Frequency	Night only; 3.5 shifts per week	Day and night; ~14 day and 7 night shifts per month	Day only; shift frequency not available	Night only; shift frequency not available	Night only; 3.5 shifts per week
Responsibilities	Direct patient care in hospital A ED	Direct patient care in ETU; assessment of patients in hospital A ED and triage area; cleaning and disinfection of grossly contaminated surfaces in hospital A triage area; cleaning and disinfection of grossly contaminated surfaces in hospital A ED	Disinfecting soiled surfaces and HCWs leaving ETU ward, but inside the ETU containment area; cleaning and disinfection of grossly contaminated surfaces in hospital A triage area	Disinfecting soiled surfaces and HCWs leaving ETU ward, but inside the ETU containment area; unknown whether cleaning and disinfection activities were performed in hospital A triage area	Direct patient care in hospital A ED
Barrier precaution equipment use in ETU	Did not work in this setting	As recommended by MSF for this setting*	As recommended by MSF for this setting*	As recommended by MSF for this setting*	Did not work in this setting
Barrier precaution equipment use in hospital A ED	Gloves were used when available; use of other equipment unknown†	Double gloves and gown reported at a minimum for all patient and cleaning encounters; use of additional mucus membrane barrier precaution equipment variable†	Unknown	Unknown	Gloves were used when available; use of other equipment unknown†
Ill contacts outside of work	Unknown	None reported	None reported	Unknown	Unknown
Date of symptom onset	July 14	July 22	July 22	July 23	July 29
Outcome	Died July 26	Recovered	Recovered	Died July 27	Recovered
Case status	Laboratory confirmed§	Laboratory confirmed§	Laboratory confirmed§	Probable	Laboratory confirmed§
Additional comments	No other HCWs in cluster were reported to have contact with this HCW after July 14	Participated in cleaning and disinfecting surfaces grossly contaminated on July 14	No additional information	Died with hemorrhagic manifestations of EVD	Had direct, unprotected patient contact with undetected, but infected patient in hospital A ED on July 14
	Did not work on July 14				Never worked same night shift as HCW A
Information source	Indirect: interview of coworkers, administrators; review of work schedule	Direct: interview Indirect: interview of coworkers, administrators; review of work schedule	Direct; interview Indirect: interview of coworkers, administrators; review of work schedule	Indirect: interview of coworkers and administrators; review of work schedule	Indirect: interview of coworkers and administrators; review of work schedule

**Abbreviations:** ED = emergency department; MSF = Médecins Sans Frontières (Doctors Without Borders).

* A description of personal protective equipment use recommended for ETUs can be found in Sterk E. Filovirus haemorrhagic fever guidelines, Médecins Sans Frontières, 2008:34. Available at http://www.slamviweb.org/es/ebola/fhffinal.pdf.

† This is not adequate barrier precaution use for caring for patients with Ebola or for cleaning and disinfecting surfaces grossly contaminated with Ebola-containing fluids.

§ Laboratory-confirmed by reverse transcription–polymerase chain reaction.

## Footnotes

* Available at http://www.cdc.gov/vhf/ebola/hcp/index.html.

† Additional information available at http://www.cdc.gov/vhf/ebola/symptoms.

§ Available at http://www.cdc.gov/vhf/ebola/hcp/infection-prevention-and-control-recommendations.html.
